# New World Health Organization guidance helps protect breastfeeding as a human right

**DOI:** 10.1111/mcn.12491

**Published:** 2017-08-10

**Authors:** Laurence M. Grummer‐Strawn, Elizabeth Zehner, Marcus Stahlhofer, Chessa Lutter, David Clark, Elisabeth Sterken, Susanna Harutyunyan, Elizabeth I. Ransom, on behalf of the WHO/UNICEF NetCode

**Affiliations:** ^1^ World Health Organization Genève Switzerland; ^2^ Helen Keller International Washington DC USA; ^3^ School of Public Health University of Maryland College Park MD USA; ^4^ UNICEF New York NY USA; ^5^ International Baby Food Action Network (IBFAN) Ontario Canada; ^6^ Yerevan State Medical University Yerevan Armenia

**Keywords:** breastfeeding, breast‐milk, breast‐milk substitutes, infant and child nutrition, infant formula, nutrition

## Abstract

Written by the WHO/UNICEF NetCode author group, the comment focuses on the need to protect families from promotion of breast‐milk substitutes and highlights new WHO Guidance on Ending Inappropriate Promotion of Foods for Infants and Young Children. The World Health Assembly welcomed this Guidance in 2016 and has called on all countries to adopt and implement the Guidance recommendations. NetCode, the Network for Global Monitoring and Support for Implementation of the International Code of Marketing of Breast‐milk Substitutes and Subsequent Relevant World Health Assembly Resolutions, is led by the World Health Organization and the United Nations Children's Fund. NetCode members include the International Baby Food Action Network, World Alliance for Breastfeeding Action, Helen Keller International, Save the Children, and the WHO Collaborating Center at Metropol University. The comment frames the issue as a human rights issue for women and children, as articulated by a statement from the United Nations Office of the High Commissioner for Human Rights.

## INTRODUCTION

A recently published statement by United Nations' experts said “Breastfeeding is a human rights issue for both the child and the mother” (United Nations Office of the High Commissioner for Human Rights, 2016). It declares that children have the right to life, survival, and development and to the highest attainable standard of health, as well as to safe and nutritious foods. Breastfeeding must be considered an integral component of these rights. Breastfeeding is also a rights issue for women (Galtry, 2015). A mother is not obligated to breastfeed her child, but no one may interfere with a mother's right to breastfeed her child (Kent, 2006). Women have the right to accurate, unbiased information needed to make an informed decision about breastfeeding and the right to an environment that enables them to carry it out. The importance of breastfeeding is now widely understood. It reduces child mortality, increases child cognition, improves maternal and child health, and fosters economic development (Victora et al., 2016).

The UN statement pointed out that promotion of breast‐milk substitutes (BMS) 1“A breast‐milk substitute should be understood to include any milks (or products that could be used to replace milk, such as fortified soy milk), in either liquid or powdered form that are specifically marketed for feeding infants and young children up to the age of 3 years (including follow‐up formula and growing‐up milks)” (WHO, 2016). by manufacturers and lack of corporate accountability for the adverse consequences of such practices pose a major obstacle to breastfeeding. The retail value of the infant formula market is enormous and rapidly growing. Estimated at US$44.8 billion in 2014, it is projected to increase to US$70.6 billion by 2019 (Rollins et al., 2016). Research shows widespread BMS promotion globally. In Cambodia, 77% of mothers saw BMS ads on television (Pries et al., 2016). In Senegal, 35% of stores selling infant foods had BMS promotions (Champeny et al., 2016). Half of mothers in Nepal reported receiving a recommendation from a health worker to give a BMS (Pries et al., 2016). Reports from the International Baby Food Action Network have documented numerous misleading nutrition and health claims attempting to create perceptions that BMS are “close to breast‐milk” (Yeong, 2016). New studies also show that milks designed for older children are being heavily promoted, frequently in ways intended to market the same brand's infant formulas (Pereira et al., 2016). Such marketing tactics provide a substantial barrier to improving breastfeeding and young child feeding practices, and to reducing under‐five child mortality and ending malnutrition (Baker et al., 2016).

What can we do to safeguard women and children's rights and protect them from such misleading practices? The 69th World Health Assembly took an important step toward further protecting women and children's rights when it adopted a resolution that calls on countries to implement the World Health Organization (WHO) *Guidance on Ending the Inappropriate Promotion of Foods for Infants and Young Children* (WHO, 2016). This new Guidance was developed in response to continuous lack of compliance with the International Code of Marketing of Breast‐milk Substitutes and subsequent relevant WHA resolutions (the Code) and increasing evidence that the promotion of BMS and some commercial complementary foods for infants and young children undermines optimal breastfeeding and young child feeding (WHO, 2015). Here is what is new about the Guidance:

1. Clarifies that “follow‐up formula” and “growing‐up milks” that are marketed up to the age of 36 months fall under the scope of the Code and should not be promoted.
2. States that messages on complementary foods should always include a statement on the need for breastfeeding to continue through 2 years and that complementary foods should not be fed before 6 months.
3. Says that labels and designs on complementary foods need to be distinct from those used on breast‐milk substitutes to avoid cross‐promotion.
4. Recognizes that any donations to the health care system (including health workers and professional associations) from companies marketing BMS and foods for infants and young children represent a conflict of interest and should not be allowed.
5. Emphasizes that sponsorship of meetings of health professionals and scientific meetings by companies selling BMS and foods for infants and young children should not be allowed.

The UN rights statement on breastfeeding pointed out that governments have an obligation under the Convention on the Rights of the Child and other relevant UN human rights instruments to respect, protect, and fulfill children's rights to health and to nutritious foods and women's rights to be protected from harmful interference by non‐State actors, particularly the business sector, and to have skilled support to enable them to breastfeed. Therefore, governments must take legislative action to fully adopt and implement the Guidance to ensure that infants and young children get the right nutrition and that mothers have support and access to accurate information about foods for their children. Without action, poor infant and young child feeding practices will continue to compromise maternal and child health and hold children, communities, and countries back.

**Key messages**

- Breastfeeding is a human rights issue for both children and mothers.
- The retail value of the infant formula market is enormous and rapidly growing.
- Research shows high prevalence of promotion of breast‐milk substitutes.
- The International Code of Marketing of Breast‐milk Substitutes prohibits the promotion of breast‐milk substitutes.
- New Guidance on Ending Inappropriate Promotion of Foods for Infants and Young Children welcomed by the World Health Assembly in 2016 builds on the Code and includes technical guidance and recommendations on how countries can strengthen their policies and programmes to protect mothers of children under 3 years of age from promotion of breast‐milk substitutes.

## CONFLICTS OF INTEREST

The authors declare that they have no conflicts of interest.

## CONTRIBUTIONS

LMG‐S, WHO, lead author: reviewed and provided substantial revisions on multiple versions, including reframing. EZ, HKI: contributed to writing the commentary, reviewed every version and made substantive contributions, authored some papers cited in commentary. MS, WHO: reviewed and provided substantial revisions. CL, University of Maryland: reviewed and provided substantial revisions on multiple versions. DC, UNICEF: reviewed and provided substantial revisions on several versions. ES, IBFAN: reviewed and provided substantial revisions. SH, Yerevan State Medical University: reviewed and provided input. EIR, HKI: conceptualized, contributed to writing, incorporated feedback, reviewed and revised every version using input from group.
